# Genotoxicity and Mutagenicity of Suspended Particulate Matter of River Water and Waste Water Samples

**DOI:** 10.1100/tsw.2002.206

**Published:** 2002-04-18

**Authors:** Georg Reifferscheid, Britta v. Oepen

**Affiliations:** Department of Environmental and Molecular Gentoxicity (AMMUG), University of Mainz, Obere Zahlbacher Str. 63, D-55101 Mainz, Germany

**Keywords:** genotoxicity, mutagenicity, suspended particulate matter, umu test, Ames test, river water, waste water

## Abstract

Suspended particulate matter of samples of river water and waste water treatment plants was tested for genotoxicity and mutagenicity using the standardized umu assay and two versions of the Ames microsuspension assay. The study tries to determine the entire DNA-damaging potential of the water samples and the distribution of DNA-damaging substances among the liquid phase and solid phase. Responsiveness and sensitivity of the bioassays are compared.

## INTRODUCTION

It has been well-documented that a number of hydrophobic compounds are adsorbed to suspended particulate matter (SPM) and sediments of rivers[1,2,3,4]. Among them, PAHs, HCB, PCBs, and chlorinated benzenes were found in concentrations up to 2 mg per kg dry weight[5]. At least in the case of PAHs, genotoxic, mutagenic, and carcinogenic potentials are evident[6]. Genotoxicological and carcinogenic evaluation of the remainder compounds is difficult, mainly due to their cytotoxicity in bioassays. Few data exist about the genotoxicity and mutagenicity of SPM of waste water samples[4,7], even though concentrations of suspended matter, in particular, are as high as those found in river water.

The present study shows results of genotoxicity and mutagenicity tests of SPM in river samples and samples of waste water treatment plants in Rhineland-Palatinate, Germany. The tests were carried out with the *umu* assay[8,9,10,11,12], the Ames-II assay[13], and a microsuspension version of the Ames assay developed by AMMUG. The *umu* assay is a so-called indicator test due to the fact that it detects primary DNA damage. In contrast, the Ames microsuspension versions measure base substitution and frameshift mutagenesis.

The aim of the study was to determine and compare the entire genotoxicity and mutagenicity of river water and waste water samples, since the current standardized assays provide information about only the liquid water phase, not the entire sample (including particle bound substances). Additionally, a comparison should be drawn between the distribution of genotoxicity and mutagenicity of particle bound substances of waste water samples and the liquid phase of the original sample. Last but not least, responsiveness and sensitivity of the *umu* assay and the Ames microsuspension assay were compared.

## EXPERIMENTAL METHODS/PROCEDURES

Original water samples of rivers (25 samples from Rhine, Mainz; Mosel, Palzem; Saar, Kanzem; Lahn, Lahnstein; and Selz, Ingelheim) and sewage plants (35 samples), soxhlet extracts of SPM, and PAD-IV extracts of the original water samples were used for genotoxicity and mutagenicity assays. Immediately after collection, the samples were frozen in order to adjust the conditions. Suspended matter was collected by filtration with glass fiber filters (pore size < 1 µm). After freeze-drying, the charged filters were soxhlet-extracted using acetone. Eluates were evaporated, and the remaining substances were dissolved in 100% DMSO. Aliquots of the extracts were incubated with tester bacteria in the presence and absence of S9 mixture and cofactors. PAD-IV solid phase extraction of filtrated water samples was performed according to[9].

The tester strain *Salmonella typhimurium* TA1535/pSK1002 was used for the *umu* assay. The Ames test was carried out using strain TA98 for frameshift mutagenesis, TAMix, a set of six base-specific point-mutation strains (TA7001-6), and the strain TA100 for base substitution mutagenesis. In contrast to the conventional Ames test on agar plates, the Ames-II assay and the Ames microsuspension assay by AMMUG are performed as microsuspension assays on 384-well microplates. Instead of the TA7000 line developed by Gee et al.[13], the AMMUG version uses the strain TA100 for base substitution mutagenesis, as it is recommended according to DIN 38415-4 (ISO 16240)[14,15].

## RESULTS AND DISCUSSION

None of the native waste water samples was genotoxic in the *umu* assay according to DIN 38415-3[11,12]. However, 44% of the PAD-IV extracts of the samples showed (background) genotoxicity, especially without S9 incubation. In the Ames microsuspension assay, mutagenicity could be detected in 12% of the native waste water samples. Most of the PAD-IV extracts (88%) of the original samples were mutagenic.

Both SPM of the river samples and of industrial waste water samples showed mutagenic effects in the Ames microsuspension tests. The extent of mutagenicity was quite different. Among the river samples, SPM of the Rhine showed the strongest mutagenicity. Incubation with and without S9 led to approximately the same mutagenic potentials. Especially the samples of the rivers Mosel and Saar were mutagenic when S9 was supplemented, which could be an indication of the predominance of premutagenic polycyclic aromatic hydrocarbons in these samples. Generally, extracts of SPM of industrial waste water showed stronger mutagenicity compared to SPM of river water. The SPM-associated mutagenicity load of waste water and river water differed up to 10,000-fold. Of the waste water SPMs, 97% showed a mutagenic potential in the Ames microsuspension assay (77% -S9; 89% +S9), whereas 44% were genotoxic in the *umu* assay (44% -S9; 33% + S9).

In general, correlations between mutagenicity and concentrations of polycyclic hydrocarbons of river SPM could not be detected, probably because the multitude of compounds extracted by acetone does not allow such a simplified assumption. Since a considerable amount of mutagenicity was detected without S9 incubation, not just hydrophobic substances (e.g., PAHs) account for the genotoxic effects. There is only limited knowledge about direct-acting genotoxins bound to SPM of river water and waste water samples and about the bioavailability of these substances in general.

In the large majority of cases, frameshift mutations dominated over base change mutations. The strains of the TA7000 line used as a mixture showed only marginal reactions when exposed to the extracts and provided no advantages over the base substitution strain TA100. Because a dominance of frameshift mutations is less plausible, particularly as there is no predominance of this type of mutation in screenings of chemical substances, theoretical considerations as well as analysis of literature data indicate that reasons for varying sensitivity between frameshift and base substitution mutagenesis are in different mechanisms of the generation of mutations. In strain TA98, at least 65 nucleotides of the *his* gene can be a target for frameshift mutations[16], whereas the TA7000 strains and strain TA100 require a base substitution at one distinct position of the target gene[17]. In general, the Ames microsuspension assay is appropriate for the investigation of complex environmental samples such as suspended matter. Regarding cost-effectiveness and handling, the Ames microsuspension assay has considerable advantages over the conventional Ames test. In the present study, the Ames microsuspension assay was more sensitive compared to the *umu* assay. This is in concordance with investigations of Vahl et al., who found high mutagenic potency beside weak SOS induction in extracts of SPM of the river Elbe[18]. In contrast to plate incorporation tests, the Ames microsuspension assay allows direct comparisons of sensitivity with other test systems like the *umu* assay, because definite concentrations of samples (and not doses) are used. Most bacterial indicator tests like the *umu* assay are based on the induction of DNA damage inducible genes of the SOS operon[19]. Despite their ability to quickly detect genotoxins and potential mutagens, the measurement of SOS induction cannot be equated with mutagenicity. Some chemical mutagens exert their mutagenic potential independently from error-prone repair pathway responsible for SOS mutagenesis and therefore cannot be detected by indicator tests based on the SOS system, which could be one reason for lower sensitivity. In contrast to that, the microsuspension versions of the Ames test detect mutagenicity as a consequence of error-prone dependent and independent genotoxicity.

## CONCLUSIONS

The current DIN and ISO standards of procaryotic genotoxicity and mutagenicity assays are not able to detect the entire genotoxicity/mutagenicity load of water samples, as they merely consider the liquid phase. This study showed that less than 50% of the genotoxic potential of the water samples could be assigned to the liquid phase. From our point of view, this study illustrates the necessity for intensifying investigations concerning genotoxicity and mutagenicity of suspended matter, for identifying responsible substances by effect-related chemical analysis, and for studying their bioavailability.

## ACKNOWLEDGMENTS

This paper was presented at the CSIC/ESF Workshop on Analysis, Toxicity and Biodegradation of Organic Pollutants in Groundwater from Contaminated Land, Landfills and Sediments, Barcelona, Spain, 8-10 November 2001.
